# Supplementary figures and images for: Structural and functional properties of a plant NRAMP-related aluminum transporter
Source: eLife. 2023 Apr 19;12:e85641. doi: 10.7554/eLife.85641 (PMC10115441; doi:10.7554/eLife.85641)

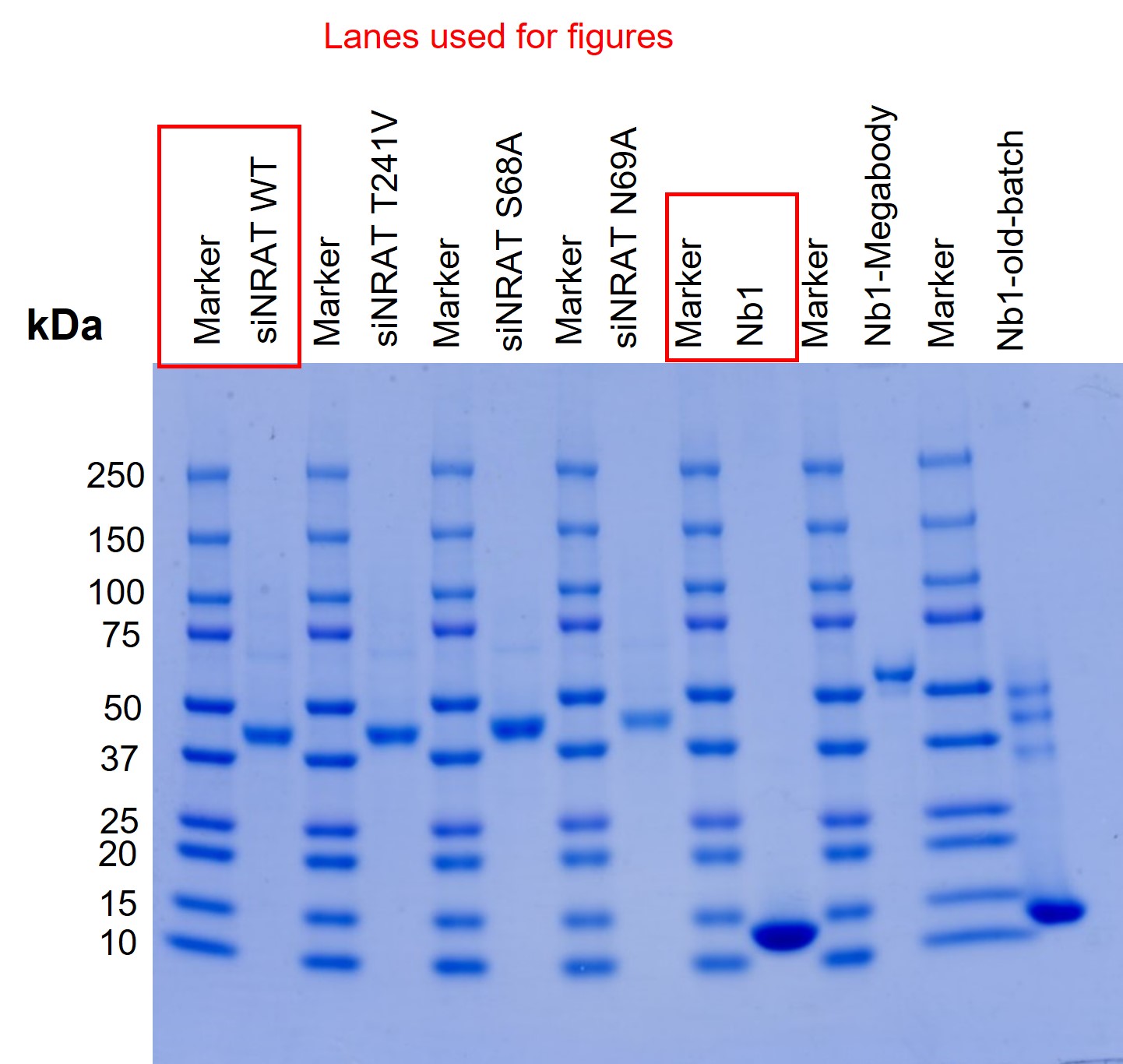

Supplement: Figure 1—figure supplement 2—source data 1. [file elife-85641-fig1-figsupp2-data1.zip › Figure 1-figure supplement 2-source data/Figure1-suppl2A_Figure2_suppl1B_SDS-PAGE-labelled.jpg]

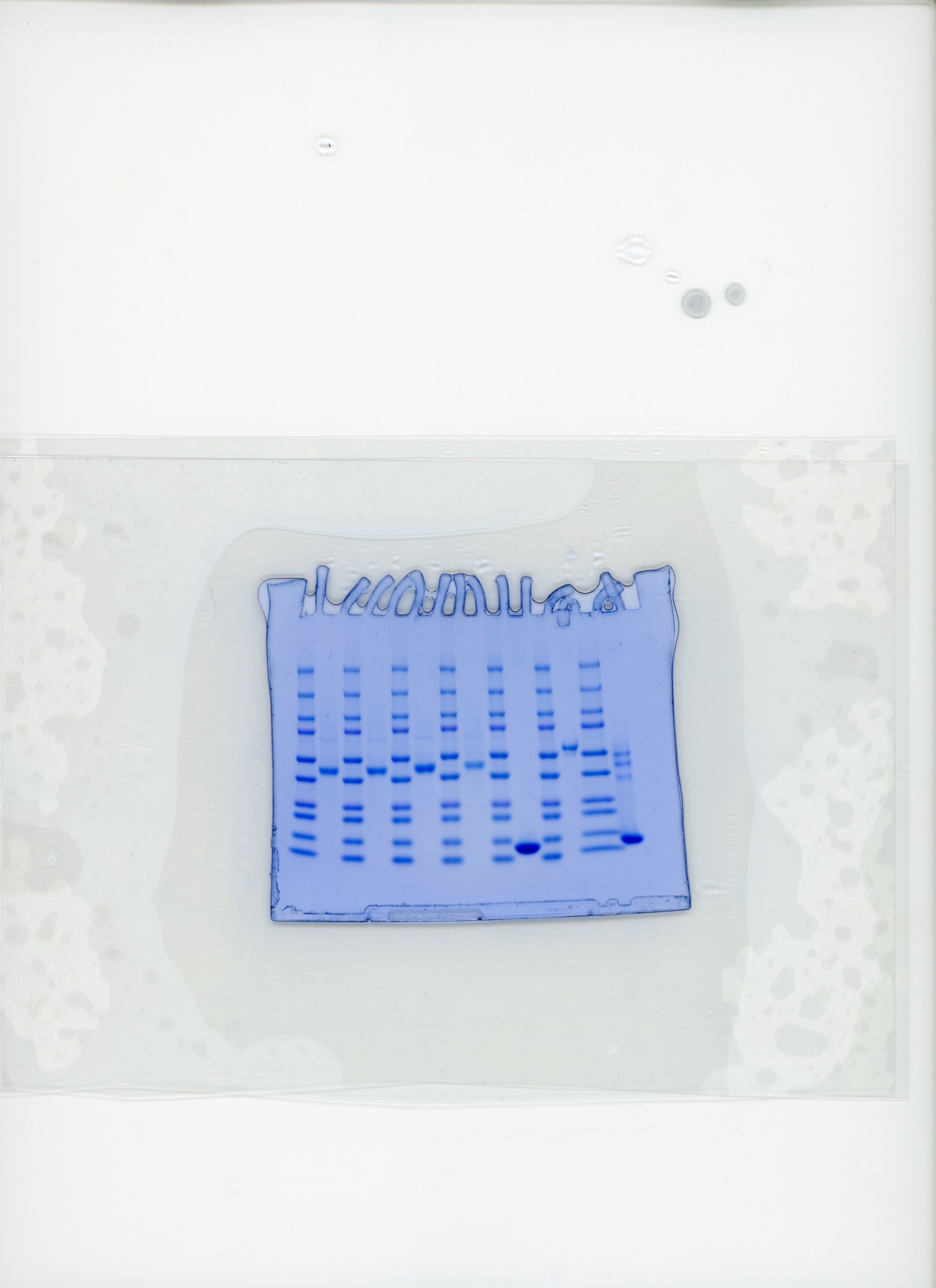

Supplement: Figure 1—figure supplement 2—source data 1. [file elife-85641-fig1-figsupp2-data1.zip › Figure 1-figure supplement 2-source data/Figure1-suppl2A_Figure2_suppl1B_SDS-PAGE-unedited.jpg]
